# Growth and safety of infants with cow’s milk allergy receiving a new hydrolyzed rice protein-based formula containing 2-fucosyllactose and lacto-N-neotetraose: protocol for a randomized clinical trial. The RIGHT-GO study

**DOI:** 10.3389/fnut.2026.1784490

**Published:** 2026-04-24

**Authors:** Boutaina Zemrani, Nicholas P. Hays, Noura Darwish, Kirsten Beyer, Roberto Berni Canani, Hania Szajewska, the RIGHT-GO Consortium, Agnieszka Brzozowska, Susanna Esposito, Urszula Jedynak-Wasowicz, Bartosz Korczowski, Diego Peroni, Anna Płoszczuk, Aleksandra Olechowska-Grad, Malgorzata Arciszewska

**Affiliations:** Department of Paediatrics and Allergy, Copernicus Memorial Hospital, Medical University of Lodz, Lodz, Poland.; Pediatric Clinic, Department of Medicine and Surgery, University Hospital of Parma, Parma, Italy.; Department of Pediatrics, Jagiellonian University Medical College, Kraków, Poland.; Department of Paediatrics and Paediatric Gastroenterology, Medical College, University of Rzeszów, Rzeszów, Poland.; Department of Clinical and Experimental Medicine, section of Pediatrics, University of Pisa, Pisa, Italy.; Prywatna Praktyka Lekarska Gabinet Pediatryczno-Alergologiczny, Bydgoszcz, Poland.; Alergo-Med Ośrodek Badań Klinicznych Sp. z o.o, Tarnów, Poland.; Poliklinika Ginekologiczno-Położnicza Arciszewscy, Bialystok, Poland.; 1 Scientific Translation and Clinical Strategy Unit, Société des Produits Nestlé, Vevey, Switzerland; 2 Biostatistics, Clinical Research Unit, Nestlé Research, Société des Produits Nestlé, Lausanne, Switzerland; 3 Department of Paediatric Respiratory Medicine, Immunology and Critical Care Medicine, Charité - Universitätsmedizin Berlin, Berlin, Germany; 4 German Center for Child and Adolescent Health (DZKJ), Partner Site Charité Universität Berlin, Berlin, Germany; 5 Pediatric Allergy Program at the Department of Translational Medical Science, University of Naples "Federico II", Naples, Italy; 6 ImmunoNutrition Lab at CEINGE Advanced Biotechnologies, University of Naples "Federico II", Naples, Italy; 7 NutriTechLab at the University of Naples “Federico II”, Naples, Italy; 8 European Laboratory for the Investigation of Food-Induced Diseases, University of Naples "Federico II", Naples, Italy; 9 Task Force for Microbiome Studies, University of Naples "Federico II", Naples, Italy; 10 Department of Pediatrics, The Medical University of Warsaw, Warsaw, Poland

**Keywords:** children, cow’s milk allergy, growth, human milk oligosaccharides, infant formula, rice, safety

## Abstract

Hydrolyzed rice formulas (HRF) are recommended by expert societies as a suitable option for the management of non-breastfed infants with cow’s milk allergy (CMA). There is a growing trend towards the use of HRF driven by their plant-based source, appealing taste, and established safety and efficacy. Human milk oligosaccharides (HMOs) play a significant role in promoting gut and immune health of infants. There is currently no evidence regarding the growth and safety of infants receiving HRF containing two HMOs. The aim of the RIGHT-GO study is to assess the growth, safety and health-related quality of life of infants receiving a new HRF containing two manufactured HMOs, 2-fucosyllactose (2’FL) and lacto-N-neotetraose (LNnT). RIGHT-GO is a randomized controlled blinded multi-center trial (NCT06633250) conducted in Europe. Healthy full-term formula-fed infants ≤ 8 months of age with physician-diagnosed or suspected CMA will be randomized to receive either the new HRF (HRF-HMO) or an extensively hydrolyzed formula (eHF-HMO) for 4 months. Both formulas contain 1.5 g/L of 2’FL and LNnT (2:1 ratio). The primary objective is to demonstrate that infants with CMA fed the new HRF-HMO have age-appropriate growth that is non-inferior to that of infants with CMA fed a control eHF-HMO. The sample size required to demonstrate non-inferiority in weight-for-age z-score with a non-inferiority margin of −0.5 standard deviation is 104 infants (52 per group). Secondary outcomes include other growth parameters (length, head circumference, bone length), allergy symptoms, gastrointestinal tolerance, health-related quality of life, as well as adverse events and formula compliance. Assessments will take place at enrollment, then monthly until 4 months post-enrollment. The RIGHT-GO study will provide strong evidence on the growth, safety and gastrointestinal tolerance of the new HRF-HMO in infants with physician-diagnosed or suspected CMA. The results will be presented at scientific meetings and published in international peer-reviewed journals.

**Clinical trial registration:**

https://clinicaltrials.gov/study/NCT06633250, Identifier: NCT06633250.

## 1 Introduction

Cow’s milk allergy (CMA) is one of the most common food allergies in childhood, with an estimated global prevalence of 0.5 to 7.5% (1–3). Clinical manifestations of CMA range from mild to severe and include cutaneous, gastrointestinal, respiratory and general symptoms (4). Breastmilk is the ideal source of nutrition for all children including for those with CMA (1). However, in situations where breastfeeding is not possible, it is essential to utilize a suitable hypoallergenic formula.

Historically, extensively hydrolyzed formulas (eHF) and amino acid-based formulas (AAF) were utilized for infants diagnosed with CMA. In the last two decades, hydrolyzed rice formula (HRF) were developed, and are now endorsed by most expert societies, such as the European Society of Pediatric Gastroenterology, Hepatology and Nutrition (ESPGHAN), as a suitable plant-based alternative to cow’s milk-derived eHF for the dietary management of CMA when breastfeeding is not possible (1). The World Allergy Organization Diagnosis and Rationale for Action against Cow’s Milk Allergy (DRACMA) and the Nutrition Committee of the French Society of Pediatrics also recommend using an extensively cow’s milk-based hydrolyzed formula or a hydrolyzed rice formula as the first option for managing non-breastfed infants with immunoglobulin E (IgE) and non-IgE-mediated CMA (2, 5). On the other hand, the Global Allergy and Asthma European Network (GA^2^LEN) Task Force makes no recommendation for or against hydrolyzed plant-based formulas for managing food allergy in infancy (6). The use of HRF has been limited by geographical availability and healthcare professionals awareness (7), but HRF are increasingly accessible today, and a growing body of evidence is being generated to support their use.

Rice is a valuable source of protein providing several essential amino acids (4). HRF are generally fortified with limiting amino acids such as lysine and threonine to fulfill the amino acid requirements of children. The protein derived from rice is hydrolyzed to enhance its water solubility (4). Rice is a cereal with low allergenicity and absence of cross-reactivity with cow’s milk proteins, making rice-based formulas an appropriate alternative for children with CMA (4, 8, 9). There have been concerns regarding the arsenic content in rice, however arsenic levels in HRF are rigorously tested and maintained within the safe range stipulated for ‘food for special medical purposes intended for infants and young children’ (FSMP) in the European Union framework (EU 2023/915 of 25 April 2023) (10, 11).

The most appealing attributes of HRF are their plant-based source and palatability. There is a growing trend towards using plant-based sources of protein in the diets of modern societies including for children. Additionally, HRF may have better palatability compared to eHF and AAF (12), offering families a broader range of options for the nutritional management of non-breastfed children with CMA. Moreover, HRF are gaining popularity among healthcare professionals due to their proven efficacy and safety, comparable to eHF, and high acceptability among consumers. Based on available evidence, rice formulas without HMOs have been shown to be safe, well tolerated and hypoallergenic in children with CMA (7). Clinical studies have shown that feeding formulas based on rice-protein hydrolysates ensures satisfactory growth from the first weeks of life up to 24 months of follow-up and supports good gastrointestinal tolerance both in healthy children (13, 14) and in those with CMA (15–22). However, HRF have been less studied than cow’s milk-based eHFs, and data from large, well-conducted randomized clinical trials is limited (1).

Human milk oligosaccharides (HMOs) are the third largest solid component of human milk but are almost absent from cow’s milk (23). HMOs are a group of a diverse and complex oligosaccharides that support gut microbiota and immune health (24), with 2-fucosyllactose (2’FL) and lacto-N-neotetraose (LNnT) being among the most abundant HMOs (25). The addition of 2’FL and LNnT to an eHF formula have partially corrected the dysbiosis commonly seen in infants with CMA (26), and importantly they were safe and associated with a lower infection rate and a lower risk of antibiotic use in children with CMA (27). Manufactured HMOs are increasingly added to infant formulas owing to their expected benefits, yet their addition to HRF remains scarce. Only one small single-arm study has investigated the growth and tolerance of a HRF containing 2’FL in 27 infants with feeding intolerance symptoms or suspected CMA and showed a good safety profile (21). To our knowledge, no study has tested the combination of a HRF with 2’FL and LNnT in children with CMA.

The purpose of this study is to determine whether a new HRF containing two manufactured HMOs (HRF-HMO) is safe and well-tolerated by infants with CMA. The primary objective is to demonstrate that infants with CMA fed the HRF-HMO have age-appropriate growth that is non-inferior to that of infants with CMA fed a control eHF with manufactured HMOs (eHF-HMO). Secondary objectives are to evaluate other growth metrics, gastrointestinal tolerance, allergy-related symptoms, health-related quality of life, and formula acceptability and palatability.

## 2 Methods and analysis

### 2.1 Study design and setting

The RIGHT-GO study is a randomized, blinded, non-inferiority trial with a parallel arm design. It will involve participation from 19 centers across three countries: Poland, Italy, and Germany. The study will be conducted by investigators specializing in pediatric allergy, pediatric gastroenterology, and general pediatrics in academic and private institutions. The study (Protocol version 1.0, dated 20 February 2023) was approved by ethical committees of all participating sites, and all procedures will be conducted according to the Helsinki Declaration.

**Figure 1:**
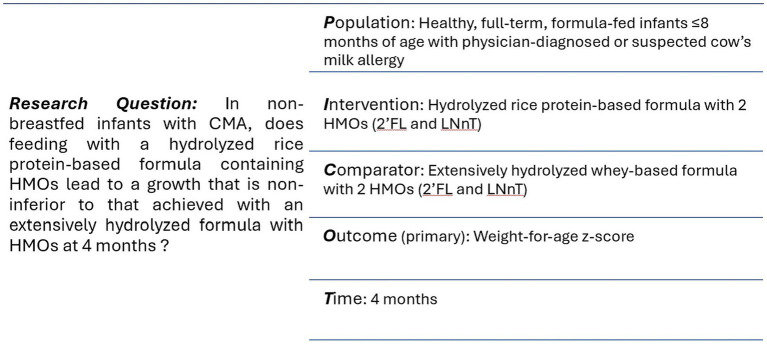
PICOT framework. CMA, cow’s milk allergy; HMO: human milk oligosaccharides; 2’FL, 2-fucosyllactose; LNnT, lacto-N-neotetraose.

**Figure 2:**
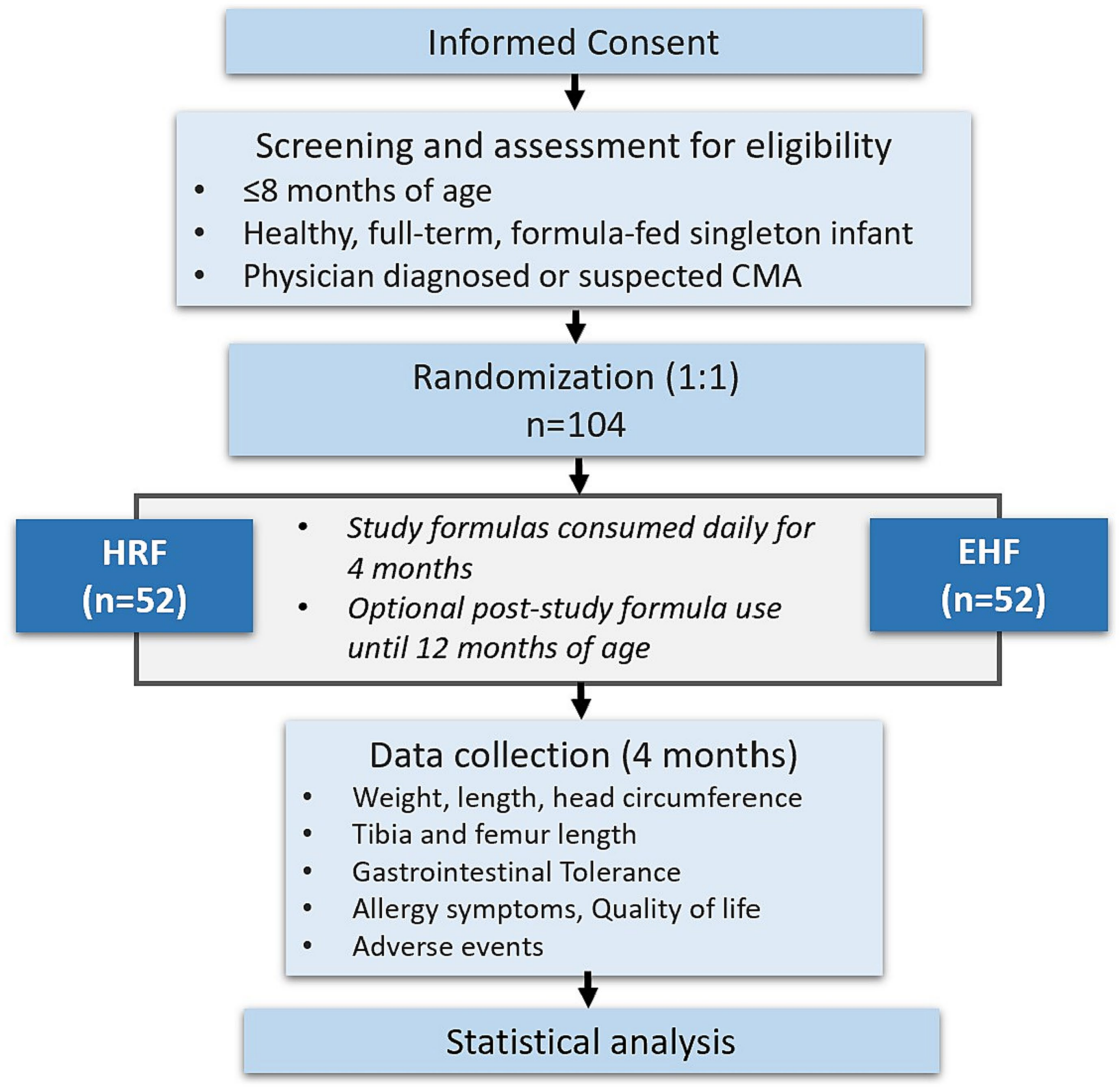
Study flow chart. CMA, cow’s milk protein allergy; EHF, extensively hydrolyzed formula; HRF, hydrolyzed rice formula.

The acronym ‘RIGHT-GO’ stands for ‘Rice-based Infant formula Growth and Hypoallergenicity Trials.’ This initiative encompasses two key studies: a growth and safety study, known as ‘RIGHT-GO study’ and a hypoallergenicity study referred to as ‘RIGHT-HY study’. Figures 1, 2 summarize the PICOT framework and design of the RIGHT-GO study.

### 2.2 Selection and treatment of infants

Term, singleton infants ≤ 8 months of age who are formula-fed at the time of enrollment, and who have physician-diagnosed or suspected CMA will be eligible for enrollment. Suspected CMA is defined as per standard clinical practice and in conjunction with at least two of the following symptoms: inconsolable crying, regurgitation, liquid stools or constipation, skin atopic lesion, cow’s milk provoked temporary urticaria/angioedema or vomiting, bloody streaks in stool, or respiratory symptoms. If an infant has been diagnosed with CMA based on either a food challenge, positive IgE blood test or skin prick test, only one other symptom is required. Parents should be willing to feed their infants a cow’s milk elimination diet throughout the study.

Infants who have a history of intolerance to eHF or rice protein allergy will be excluded as well as infants with chronic medical disease (except atopic eczema), major gastrointestinal disease, known or suspected lactose intolerance or malabsorption, glucose-galactose malabsorption or soy allergy. In addition, infants with or history of severe anaphylaxis to cow’s milk or breastmilk prior to enrollment will be excluded. Infants with a weight-for-age or height-for-age z-score <−2 or >2 on the World Health Organization (WHO) Growth Standards at enrollment will also not be eligible for enrollment.

#### 2.2.1 Sample size

The sample size calculation is based on the reported weight-for-age z-score after 4 months of formula feeding in a similar study in infants with CMA (27). To demonstrate non-inferiority with 90% power, a non-inferiority margin of - 0.5 z-score and *α*-level of 0.05, 36 infants would be needed per group. This calculation assumes that the control group would have growth rate that is 0.03 z-score slower than the test group due to a slightly lower protein content in the control formula. Assuming a drop-out rate of 30%, a total sample size of 104 infants (52 infants per group) will need to be recruited to have 72 completers (36 per group).

#### 2.2.2 Randomization, allocation and blinding

Upon obtaining informed consent, infants will be randomized to Test or Control Formula in a 1:1 ratio. Randomization will be carried out by minimization with center, sex, and age category (0 to ≤60 days, 60 to ≤ 120 days, 120 to ≤ 180 days, > 180 days) as stratification factors, using the dynamic allocation 2nd Best algorithm provided by Medidata Rave ‘Randomization and Trial Supply Management’ (RTSM) technology. The second-best probability parameter (i.e., the probability that Rave RTSM assigns a subject to one of the second-choice study group assignments) will be set at 15% to reduce the deterministic nature of the minimization algorithm.

The identity of the products will be blinded to infants’ parents/caregivers, investigators, site personnel, the contract research organization managing the study, and the sponsor (except the manufacturing site, supply, and quality managers). The products will be packaged and labelled identically but will be distinguishable by unique codes affixed on the primary packaging. An individual coding of cartons will be used to prevent from unblinding in case of emergency unblinding. This latter may be requested by investigators in case of serious adverse events suspected to be related to the investigational product and requiring knowledge of the study product administered.

### 2.3 Interventional methods

#### 2.3.1 Test formula: HRF-HMO

The Test formula is a hydrolyzed rice protein-based formula, without lactose, containing two manufactured HMOs structurally identical to human milk oligosaccharides, 2’FL and LNnT, at 1.5 g/L in a 2:1 ratio. These HMO levels were chosen to be close to those found in human milk. The protein content is 2.3 g/100 kcal with a Protein Digestibility-Corrected Amino Acid Score (PDCAAS) of 1. The arsenic content of the formula is maintained within safe limits, below the maximum inorganic arsenic content for FSMP intended for infants and young children under the age of 3 set by the European commission (Regulation EU 2023/915 of 25 April 2023) (28). The macronutrient and HMO composition of the study formulas is presented in Table 1.

**Table 1:** Macronutrient and HMO composition of study formulas.

Formula composition	Hydrolyzed rice protein-based formula (HRF-HMO)	Extensively hydrolyzed whey-based formula (eHF-HMO)
Caloric density (kcal/100 mL)	67.5	66.5
Protein (g/100 kcal)	2.3*	2.20
Protein (g/100 mL)	1.55	1.46
Protein source	Hydrolyzed rice protein	Extensively hydrolyzed whey
Lipids (g/100 kcal)	5.2	5.2
Carbohydrate (g/100 kcal)	10.8	11.1
Carbohydrate source	Maltodextrin, starch	Maltodextrin, lactose
2’FL (g/L)	1.0	1.0
LNnT (g/L)	0.5	0.5

EHF, extensively hydrolyzed formula; HRF, hydrolyzed rice formula; HMO, human milk oligosaccharides; 2’FL, 2-fucosyllactose; LNnT, lacto-N-neotetraose.*minimum protein value of 2.25 g/100 Kcal.

#### 2.3.2 Control group: eHF-HMO

The control formula is a commercially available extensively hydrolyzed whey-based formula (Althéra® HMO, Nestlé Health Science, Vevey, Switzerland) with lactose, containing the same type and amount of HMOs as the Test formula. The protein content of the Control formula is 2.2 g/100 kcal. This formula was selected as a control as eHF are the standard of care for non-breastfed infants with CMA. Furthermore, this formula contains the same HMOs as the test formula.

The Test and Control formulas are both considered ‘FSMPs’ intended for infants with CMA. Both formulas are nutritionally complete FSMPs and fall in the scope of Delegated Regulation (EU) 2016/128. The formulas will be provided in powder form and administered orally after reconstitution with water, using an infant feeding bottle, cup, bowl, or other container depending on age and developmental stage. Instructions for proper study formula handling and preparation will be provided to parents. Minor taste differences exist between the two formulas due to their inherent composition.

Study formulas will be administered for 4 months, and infants will be fed daily *ad libitum.* The volume of formula required each day may vary depending on the infant’s age, weight, and appetite. After 4 months of feeding the assigned study formula, parents will be offered post-study access to their assigned formula until the child is 12 months of age. This continued access to the study formula is intended to ensure the health and well-being of all study participants who may still need access to a hypoallergenic formula that has been identified as beneficial for that participant during the study.

### 2.4 Data analysis

#### 2.4.1 Primary outcome

The primary outcome is weight-for-age z-score after 4 months of intervention. The treatment difference between the Test formula and Control formula will be estimated by a linear mixed-effect model. Fixed effects will include baseline weight-for-age z-score, age at baseline (0 to ≤ 60 days, 60 to ≤ 120 days, 120 to ≤ 180 days, > 180 days) and center. Random effect will be the infant. Primary analysis will be in the per-protocol (PP) dataset. Weight-for-age z-score after 4 months of test formula intake will be at least as good as the control, if the treatment difference between the Test group (HRF-HMO) and Control group (eHF-HMO) is < −0.5 SD (non-inferiority margin) (29). The treatment difference is superior to −0.5 SD if the lower bound of the two-sided 95% confidence interval is more than −0.5 SD. Such procedure corresponds to a one-sided testing at 2.5% level.

#### 2.4.2 Secondary outcomes

##### 2.4.2.1 Growth assessment

Growth outcomes include weight-for-age, length-for-age, head circumference-for-age, weight-for-length and BMI-for-age z-scores, as well as tibia and femur length. Weight and length will be collected at enrollment and monthly throughout the study, while head circumference, tibia and femur length will be measured at baseline and at 4 months post-intervention. Anthropometry parameters will be measured using standardized methodology and calibrated instruments by trained personnel. Weight, length and head circumference measurements will be performed two times by two trained persons, to the nearest 10 g, 0.1 cm and 0.1 cm respectively; they will then be plotted against the WHO Growth Standards. Tibia length corresponds to the distance between the tip of the medial condyle and the tip of the medial malleolus. Femur length is the distance between the tip of the greater trochanter and the lowest point of the lateral condyles. Both measurements should be taken three consecutive times, to the nearest 0.1 cm in a lying position using a tape measure.

Considering that complementary food intake may affect growth outcomes, dietary intake will be evaluated through 24-h dietary recalls conducted at baseline, as well as at the 2 and 4-month visits. This assessment aims to determine the type and quantities of all foods and beverages consumed by the infant. Investigators will receive detailed instructions and training to ensure accurate recording of complementary food intake.

##### 2.4.2.2 Gastrointestinal tolerance

To assess gastrointestinal (GI) tolerance to study formulas, the Infant Gastrointestinal Symptom Questionnaire (IGSQ) will be administered at each visit and via a phone call after the first week of study formula use. IGSQ is a validated questionnaire including 5 symptom clusters, yielding a 13-item index of parent-reported infant digestion and elimination behaviors over the prior 7 days (30). It is the most commonly used tool to assess infant GI-related symptoms in clinical studies. During the first week of the study period, a daily gastrointestinal tolerance questionnaire will also be completed by the parents, including questions on the presence and severity of flatulence, vomiting, crying, fussing and sleep duration.

The Brussels Infant and Toddler Stool Scale, a validated stool scale, will be used to assess stool consistency (31). Parents will document stool frequency and consistency (categorized as watery, loose, formed, or hard) in a diary over three consecutive days prior to the 2- and 4-month visits, along with a one-day retrospective record of stool frequency and consistency at baseline.

##### 2.4.2.3 Allergy symptoms

Assessment of allergy symptoms will be conducted at baseline and each visit using the Cow’s Milk-related Symptom Score (CoMiSS) (32). CoMiSS is a validated awareness tool for CMA and includes possible manifestations of CMA such as skin, GI, respiratory and general symptoms. CoMiSS can also be used to evaluate and quantify the evolution of allergy symptoms, with scores ranging from 0 to 33.

##### 2.4.2.4 Health-related quality of life assessment

Health-related quality of life assessment will be measured at baseline and at the 4-month visit using validated questionnaires. Parental quality of life will be assessed using the Food Allergy Quality of Life Questionnaire, parent form (FAQLQ-PF) (33), and parental self-efficacy in managing child’s food allergy will be evaluated using the Food Allergy Self-Efficacy Scale for Parents (FASE-P) (34). Parent-reported infant health-related quality of life will be measured using the Infant and Toddler Quality of Life Questionnaire-Short Form (ITQOL-SF47) (35).

##### 2.4.2.5 Formula compliance and liking

Compliance to study formulas will be monitored during each visit through a standardized set of questions inquiring about the volume of formula intake, any interruptions to the study formula, as well as the consumption of unauthorized formulas or complementary foods, particularly in relation to adherence to the cow’s milk elimination diet. Compliance is also verified via formula accountability at each visit by counting the number of remaining and returned cans. A ‘non-compliant day’ is defined as any day on which a formula other than the study formula was consumed, complementary foods or drinks containing cow’s milk products were ingested, or complementary feedings were introduced prior to the age of four months. A compliance threshold of 85% was established to define adequate adherence to study products. Formula liking will be assessed at the end of the study via a simple questionnaire that captures the ease of formula preparation and overall liking by the parent and child.

##### 2.4.2.6 Adverse events

Adverse events will be collected from the time informed consent is signed until the end of the 4-month intervention. Evaluation of potential adverse events will be performed during each visit and phone call by study investigators. The type, severity, seriousness, duration and relationship of the adverse events to study formulas will be recorded and listed by system-organ class and preferred term according to the Medical Dictionary for Regulatory Activities (MedDRA) glossary. The intervention with the two formulas is considered low risk and no significant adverse events are expected. During the period where parents are offered a post-study access to the assigned formula, no data will be proactively collected, however adverse events can be spontaneously reported by parents.

#### 2.4.3 Study schedule

Study visits will take place on-site at enrollment and 1, 2, 3, and 4 months post-enrollment. Additionally, a phone call to the caregivers will be made on day 7 after enrollment to inquire about the infant’s health status and the occurrence of any adverse events. A detailed overview of the study procedures can be found in Table 2.

**Table 2:** Study visits and assessments.

Visit or phone call number	V0	C1	V1	V2	V3	V4
Infant age	≤ 8 mo	V0 + 7 days (±3 days)	V0 + 1 mo (±7 days)	V0 + 2 mo (±7 days)	V0 + 3 mo (±7 days)	V0 + 4 mo (±7 days)
Informed consent	X					
Assign subject number	X					
Inclusion/exclusion criteria	X					
Physical examination	X					X
Medical history	X					
Parent / household characteristics	X					
Randomization	X					
Weight and Length	X		X	X	X	X
Head Circumference	X					X
Tibia and femur length	X					X
Infant Gastrointestinal Symptom Questionnaire (IGSQ)	X	X	X	X	X	X
Daily gastrointestinal tolerance questionnaire	→	→ |				
Retrospective 1-day stool record	X					
3-day stool diary				X		X
CoMiSS Questionnaire	X		X	X	X	X
Food Allergy Quality of Life Questionnaire	X					X
Food Allergy Self-Efficacy Questionnaire	X					X
ITQOL-SF 47	X					X
Formula compliance		X	X	X	X	X
Parent and child product liking						X
Dietary intake assessment (24-h recall)	X			X		X
Formula dispensation and accountability	X		X	X	X	X
Cow’s milk elimination diet	→	→	→	→	→	→
Concomitant medication/diet record	→	→	→	→	→	→
Adverse event record	→	→	→	→	→	→

C, call; CoMiSS, Cow’s Milk-related Symptom Score; ITQOL-SF 47, Infant and Toddler Quality of Life Questionnaire-Short Form; Mo, months; V, visit.

#### 2.4.4 Data analysis

The analyses of this study will adhere to the recommended guidelines outlined in the CONSORT 2022 statement for reporting outcomes in clinical trial reports (36).

The Intention-to-Treat (ITT) analysis population will include all randomized infants. The full analysis set (FAS) will consist of a subset of subjects from the ITT with available weight-for-age z-scores at baseline and 4 months and available measurements for all other covariates included in the model. The Per Protocol (PP) set will include all infants from the FAS having no protocol deviations impacting the primary endpoint, nor documented concomitant medication intakes impacting the primary endpoint, nor violated eligibility criteria. In particular, infants that are non-compliant to feeding interventions will be excluded from the PP set. The analysis in aforementioned populations will be conducted using the assigned intervention. The safety analysis set (SAF) will consist of all infants in the ITT population with documentation of at least one administration of study product, analyzed according to the product received irrespective of the randomization assignment. The primary and secondary endpoints will be analyzed in both FAS and PP populations.

Descriptive statistics will be calculated and tabulated for all categorical and continuous variables by visit and feeding group. The primary endpoint of weight-for-age z-score after 4 months of intervention will be analyzed using a linear mixed-effect model with baseline weight-for-age z-score, sex, age at baseline (0 to ≤ 60 days, 60 to ≤ 120 days, 120 to ≤ 180 days, > 180 days) and center included as fixed effects. Random effect will be the subject. Non-inferiority will be concluded if the lower bound of the two-sided 95% confidence interval (CI) of the mean difference in weight-for-age z-score after 4 months of intervention between Test group and Control group is more than −0.5 z-scores (non-inferiority margin). Such procedure corresponds to a one-sided testing at 2.5% level. For secondary endpoints, continuous variables (anthropometrics, IGSQ score, CoMiSS score, stool consistency and energy and macronutrient intake) will be compared between groups using a linear mixed-effect model. Stool frequency as a count variable will be compared between the groups by a generalized model approach. All secondary endpoints models will include baseline measurement, sex, age at baseline, and center as covariates. Random effect will be the subject (wherever applicable). Secondary endpoints comparisons will be performed using two-sided tests at 5% level. Occurrence of adverse events will be summarized in a table presenting frequency, severity, seriousness of adverse events, as well as their relationship to the assigned formulas.

Data will be entered from the source document into an electronic Case Report Forms by the investigators. Data quality will be regularly monitored in a blinded way by the sponsor throughout the study. Where required, queries will be sent to the sites, and all data modifications will be documented in an audit trail file.

## 3 Discussion

To the best of our knowledge, this is the first study to evaluate a HRF with two manufactured HMOs in infants with CMA. This clinical trial will provide robust clinical evidence for this new HMO-containing rice-based formula to support the growth, gastrointestinal tolerance, safety and quality of life of infants with CMA.

Growth concerns are prevalent in children with CMA, both prior to diagnosis and during elimination diet (1). Feeding difficulties, therapeutic elimination diet, along with nutrient malabsorption due to sustained intestinal inflammation might adversely affect the growth of infants with CMA (1). Achieving age-appropriate growth and adequate nutrient intake in this vulnerable population is therefore critical. Cow’s milk elimination diet may lead to micronutrient deficiencies including vitamin D and calcium, which may negatively impact bone growth and quality (37–40). Hypoallergenic formulas can enhance the quality of cow’s milk-free diet of non-breastfed infants, by improving the intake of essential nutrients including vitamin D, calcium, protein and energy (41, 42).

CMA may lead to substantial impairment in the quality of life of affected children and their caregivers. Health-related quality of life is considered one the most meaningful outcome measures for food allergies (43). Providing a formula that ensures lasting relief of allergy symptoms, while being safe and nutritionally complete, is crucial for the quality of life of both non-breastfed children and their caregivers.

Data from preclinical studies suggest that HMOs may significantly contribute to the gut and immune health of non-breastfed infants with CMA (44, 45). The addition of HMOs to standard infant formulas and to eHFs have shown evidence for benefits on gut microbiota and clinically relevant endpoints, such as the reduction in the rate of common infections and antibiotic use in non-breastfed infants (26, 27, 46, 47). While the current study examines the growth and safety of this new HRF-HMO, future research should investigate the potential beneficial impact of HMOs on gut microbiota and the frequency of common infections in children with CMA.

With the growing popularity of plant-based diets and increasing availability of HRF, the use of HRF is expected to rise, not only in infants with CMA, but possibly also in healthy infants. The findings of this study will provide prospective multicenter data on critical outcomes in infants with physician-suspected and confirmed CMA, thereby enhancing physicians’ confidence to recommend HRF. Enabling families to have treatment options that align with their social, religious or cultural beliefs can also enhance their satisfaction with the therapeutic process.

Our study has few limitations. First, it includes both physician-suspected and confirmed cases of CMA, and not only oral food challenge-proven cases. Although the latter approach represents the gold standard for diagnosis of CMA, the former more accurately reflects real-world practice and would enhance the generalizability of our findings. Second, the intervention period is relatively short; however, a 4-month period is adequate to assess the key endpoints of this study. Lastly, the study will generate evidence in the European population, however the core findings can be adapted to other contexts.

One of the main strengths of this study lies in its robust methodology characterized by a randomized controlled and blinded design. Demonstrating safety and efficacy in young children requires high-quality scientific studies. The multicentric framework involving more than 15 sites across three European countries, will also support the generalizability of the findings. Notably, to the best of our knowledge, this is the first study to evaluate the safety of a HRF containing two manufactured HMOs in infants.

## 4 Conclusion

This study is the first to date conducted with a HRF including 2’FL and LNnT in infants with physician- suspected and confirmed CMA. It will provide robust data on growth, tolerance, quality of life and safety of infants receiving this new formula, offering families of non-breastfed infants with plant-based and palatable option for managing CMA.

Future research should investigate the impact of this HRF with added HMOs on gut microbiota, bone quality and occurrence of common infections in children with CMA. Additionally, the role of HRF in children experiencing persistent CMA symptoms while on eHF, as well as in those with multiple food allergies, should be investigated.

## The RIGHT-GO Consortium

Agnieszka Brzozowska: Department of Paediatrics and Allergy, Copernicus Memorial Hospital, Medical University of Lodz, Lodz, Poland. Susanna Esposito: Pediatric Clinic, Department of Medicine and Surgery, University Hospital of Parma, Parma, Italy. Urszula Jedynak-Wasowicz: Department of Pediatrics, Jagiellonian University Medical College, Kraków, Poland. Bartosz Korczowski: Department of Paediatrics and Paediatric Gastroenterology, Medical College, University of Rzeszów, Rzeszów, Poland. Diego Peroni: Department of Clinical and Experimental Medicine, section of Pediatrics, University of Pisa, Pisa, Italy. Anna Płoszczuk: Prywatna Praktyka Lekarska Gabinet Pediatryczno-Alergologiczny, Bydgoszcz, Poland. Aleksandra Olechowska-Grad: Alergo-Med Ośrodek Badań Klinicznych Sp. z o.o, Tarnów, Poland. Malgorzata Arciszewska: Poliklinika Ginekologiczno-Położnicza Arciszewscy, Bialystok, Poland.
